# Supplementary material for: Identification of TAP2 protein variants resistant to inhibition by the HSV1 ICP47 protein
Source: bioRxiv. 2024 Nov 18:2024.11.18.624061. Preprint. [Version 1] doi: 10.1101/2024.11.18.624061 (PMC11601387; doi:10.1101/2024.11.18.624061)
Supplement: 1 [file NIHPP2024.11.18.624061v1-supplement-1.pdf]

bioRxiv preprint doi: https://doi.org/10.1101/2024.11.18.624061; this version posted November 18, 2024. The copyright holder for this preprint (which was not certified by peer review) is the author/funder, who has granted bioRxiv a license to display the preprint in perpetuity. It is made available under aCC-BY 4.0 International license.

LEGENDS TO SUPPLEMENTARY FIGURES

Figure S1. Scatterplots of flow cytometry results from U2OS cells expressing TAP2 variants for effects of ICP47 on MHC-I surface abundance.

In each scatterplot, the x-axis is the intensity of MHC-I antibody stain, and the y-axis is the intensity of dsRed stain. Uninfected cells are shown on the left, and cells expressing dsRed and ICP47 are shown on the right. Each row is a different variant of TAP2: TAP2-wild-type, TAP2-S274H, TAP2-Y537P, TAP2-T257I, TAP2-T244R, and TAP2-T499D, as indicated. Each cell line is a U2OS-derived cell with TAP2 knocked out by CRISPR and rescued by the stable integration of the indicated variant at a single recombination site in the genome.

Figure S2. Scatterplots of flow cytometry results from U2OS cells expressing TAP2 variants for effects of BNLF2a on MHC-I surface abundance.

As in Figure S1, each scatterplot measures the intensity of MHC-I antibody stain (x-axis) and dsRed (y-axis). The left column contains results from ICP47-expressing cells (a different experiment than used in Figure S1) and the right column contains results from the same cells but expressing BNLF2a.

Figure S3. Full images of immunoblots

Full exposures are shown for immunoblots shown in Figure 3C.

LEGENDS TO SUPPLEMENTARY TABLES

Table S1. Read-count frequency for each TAP2 variant in cells with high MHC-I surface stain.

TAP2 variants detected in the high MHC-I surface stain cells are listed in order of the number of read-counts for each variant. The six highest are indicated in red, the next six in yellow, and the top 39 are indicated with color highlights. The same colors are used to label the residues in the 3D structure in Figure 2C. Synonymous and nonsense variants are highlighted in gray.

bioRxiv preprint doi: https://doi.org/10.1101/2024.11.18.624061; this version posted November 18, 2024. The copyright holder for this preprint (which was not certified by peer review) is the author/funder, who has granted bioRxiv a license to display the preprint in perpetuity. It is made available under aCC-BY 4.0 International license.

Table S2. Functional scores of variants independent of a viral inhibitor protein.

The functional scores of 1405 variants were scored in prior experiments [11]. Functional scores less than 0.92 were judged loss of function, and functional scores greater than 2.2 were judged hyperfunctional. The 39 variants that were significantly enriched in the pool of TAP2 variants are shown with their functional scores and interpretation.

Table S3. Quantitation of singleton assays for TAP2 variant resistance to inhibition by ICP47 or BNLF2a

Results of the median scores for variant effects on MHC-I surface abundance are indicated for each replicate. The ratio of the median in the presence of ICP47 to the median in the absence of ICP47 was used to compare variants to wild-type and was used in the table in Figure 3B.
